# Difference between arterial and venous peak optical density after thrombectomy is associated with functional outcomes

**DOI:** 10.3389/fneur.2024.1414898

**Published:** 2024-07-05

**Authors:** Guangzhi Liu, Jianghui Cao, Peiyang Zhou, Dong Sun, Zhiming Kang, Ruixue Fan, Bin Mei, Junjian Zhang

**Affiliations:** ^1^Department of Neurology, Zhongnan Hospital of Wuhan University, Wuhan, China; ^2^Department of Neurology, Xiangyang No.1 People’s Hospital, Hubei University of Medicine, Xiangyang, China; ^3^Department of Radiology, Xiangyang No.1 People's Hospital, Hubei University of Medicine, Xiangyang, China

**Keywords:** ischemic stroke, peak optical density, cerebral reperfusion, functional outcomes, thrombectomy

## Abstract

**Background:**

The density of contrast medium in digital subtraction angiography (DSA) have been used to evaluate the cerebral circulation function. Our aim was to study the effect of difference in arteriovenous peak optical density (POD) after thrombectomy on functional outcomes.

**Methods:**

Consecutive patients with acute ischemic stroke due to large vessel occlusion who underwent thrombectomy were reviewed. We processed DSA images with ImageJ software to measure the POD of internal carotid artery (ICA) and cortical veins. The average POD of cortical veins (POD_VA_) and the POD difference between ICA and cortical veins (POD_ICA-CV_) were calculated. Primary outcome was good functional outcome (modified Rankin scale score of 0–2 at 90 days).

**Results:**

One hundred sixty-six patients were finally included in the study. Patients with good functional outcome had lower ipsilateral POD_VA_ (median [interquartile range (IQR)], 257.198 [216.623–296.631] vs. 290.944 [248.647–338.819], *p* < 0.001) and lower ipsilateral POD_ICA-CV_ (median [IQR], 128.463 [110.233–153.624] vs. 182.01 [146.621–211.331], *p* < 0.001). Multivariable logistic regression analyses showed that ipsilateral POD_VA_ (odds ratio [OR] 0.991, 95% confidence interval [CI] 0.984–0.999, *p* = 0.019) and ipsilateral POD_ICA-CV_ (OR 0.975, 95% CI 0.963–0.986, *p* < 0.001) were associated with good functional outcome. The predictive ability was significantly enhanced in the model including ipsilateral POD_ICA-CV_ (0.893 vs. 0.842, *p* = 0.027). No correlation was found between ipsilateral POD_ICA-CV_ and expanded Thrombolysis in Cerebral Infarction grades (*r* = −0.133, *p* = 0.099).

**Conclusion:**

Ipsilateral POD_ICA-CV_ is an additional indicator of cerebral reperfusion status and predicts functional outcomes after thrombectomy.

## Introduction

Thrombectomy has been identified as an effective treatment for patients with acute ischemic stroke due to large vessel occlusion (AIS-LVO) (1). However, despite the high rate of successfully reopening the blocked arteries and the use of various predictors such as arterial collaterals, initial core infarction, and penumbra tissue in patient selection, only around half of the patients actually benefit from thrombectomy (2, 3). Successful arterial recanalization is not enough to ensure adequate cerebral tissue reperfusion. The CHOICE trial has shown that using alteplase directly into the artery after successful thrombectomy can further improve blood flow in the small vessels and reduce the risk of hypoperfusion (4). In previous animal studies, downstream vascular dysfunction caused by microclots, compression, contraction, inflammation and reactive oxygen species has been verified as a major factor contributing to poor microcirculatory reperfusion, leading to the phenomenon known as “non-reflow” (5, 6). Therefore, it is necessary to evaluate the condition of the downstream vessels after thrombectomy to accurately assess the success of the procedure.

However, current methods for assessing downstream vascular function are not uniform. Even if patients show signs of poor downstream vascular function, they are less likely to receive timely treatment (5). There is a crucial need for a method that can quickly and effectively assess the status of downstream vessels after thrombectomy. One promising approach is to assess the cerebral venous outflow (VO). Since all blood in the brain eventually drains into the venous system, changes in cerebral VO can occur following a blockage in the upstream vessels. These changes have been observed on computed tomography angiography (CTA) images in patients with AIS-LVO and correlated with the hypoperfusion intensity ratio which is superior to arterial collateral in the assessment of tissue perfusion (7). After successful arterial recanalization, the microcirculatory disturbance can also be reflected by VO. The postoperative time to peak density (TTP) of contrast medium in the cortical vein has been found as an independent predictor of good functional outcome. Moreover, the arteriovenous TTP difference significantly improved the predictive ability for outcomes after thrombectomy (8). These results emphasize the effectiveness of comprehensive evaluation including arterial and venous circulation in gathering more information about the cerebral reperfusion. However, it is important to note that this method evaluates a distal branch of middle cerebral artery and a specific vein, resulting in a limited assessment of brain tissue. Furthermore, the processing of data obtained through this method requires the use of commercially available software.

To address this issue, we introduced a novel method to reflect the postoperative vascular function. We used ImageJ software to measure the venous peak optical density (POD) and the difference with arterial POD on digital subtraction angiography (DSA) images immediately after thrombectomy in patients with different prognosis. Our hypothesis is that the POD difference can serve as a reliable indicator to assess the effectiveness of cerebral reperfusion therapy and predict functional outcomes in patients with AIS-LVO.

## Methods

### Study design and patients

**Figure 1:**
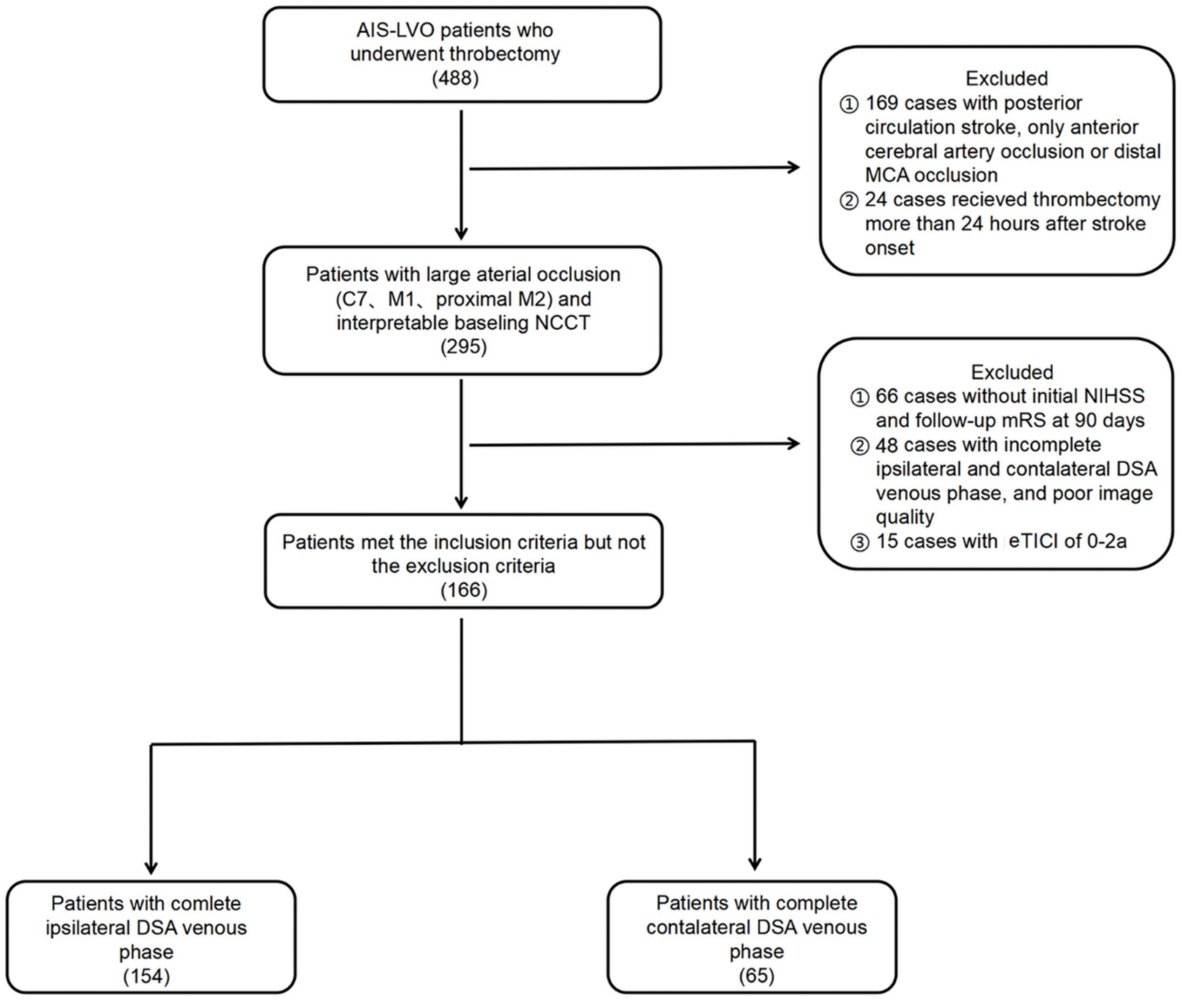
Flow chart of this study. AIS-LVO, acute ischemic stroke due to large vessel occlusion; MCA, middle cerebral artery; NCCT, nonenhanced computed tomography; NIHSS, National Institutes of Health Stroke Scale; mRS, modified Rankin Scale; DSA, digital subtraction angiography; eTICI, expanded Thrombolysis in Cerebral Infarction.

This study was approved by our institutional ethical committee. The study included consecutive patients with Acute Ischemic Stroke with Large Vessel Occlusion (AIS-LVO) who underwent thrombectomy at our center between July 2018 and July 2022. The patients were reviewed retrospectively. We included patients who met the following criteria: (1) had occlusion in the terminal internal carotid artery (ICA) or proximal middle cerebral artery (MCA); (2) received thrombectomy within 24 h after the onset of symptoms; (3) achieved successful recanalization with an expanded Thrombolysis in Cerebral Infarction grade (eTICI) of 2b-3; (4) had baseline head nonenhanced computed tomography (NCCT) images; (5) had interpretable digital subtraction angiography (DSA) images for assessing collateral circulation, arterial recanalization grades, and POD; and (6) had essential demographic and clinical data. Patients with posterior circulation or only anterior cerebral artery occlusion, a modified Rankin Scale (mRS) score of ≥2 before stroke onset, no follow-up mRS at 90 days, or poor image quality were excluded from the study. The flowchart of patient selection is shown in Figure 1.

### Demographic and clinical data

Demographic and clinical information were obtained from the electronic medical records including age, gender, medical history (hypertension, diabetes mellitus, hyperlipidemia, coronary heart disease, atrial fibrillation, smoking, drinking), initial National Institute of Health Stroke Scale (NIHSS) score, the Trial of ORG 10,172 in Acute Stroke Treatment (TOAST) classification, intravenous thrombolysis treatment (IVT), onset to puncture time (OPT), puncture to recanalization time (PRT) and onset to recanalization time (ORT).

### Imaging analysis, DSA acquisition and measurement of POD

All imaging results were independently assessed by two neuroradiologists who were blinded to the clinical information. In cases of disagreement, a third reviewer provided the final decision. The extent of baseline cerebral infarction was evaluated using the Alberta Stroke Program Early CT score (ASPECTS) on baseline nonenhanced CT images. Hemorrhagic transformation (HT) and infarct volume were evaluated within 48 h after thrombectomy using follow-up CT or magnetic resonance (MR) images. The infarct volume was measured using ITK-SNAP software version 3.8.0.

**Figure 2:**
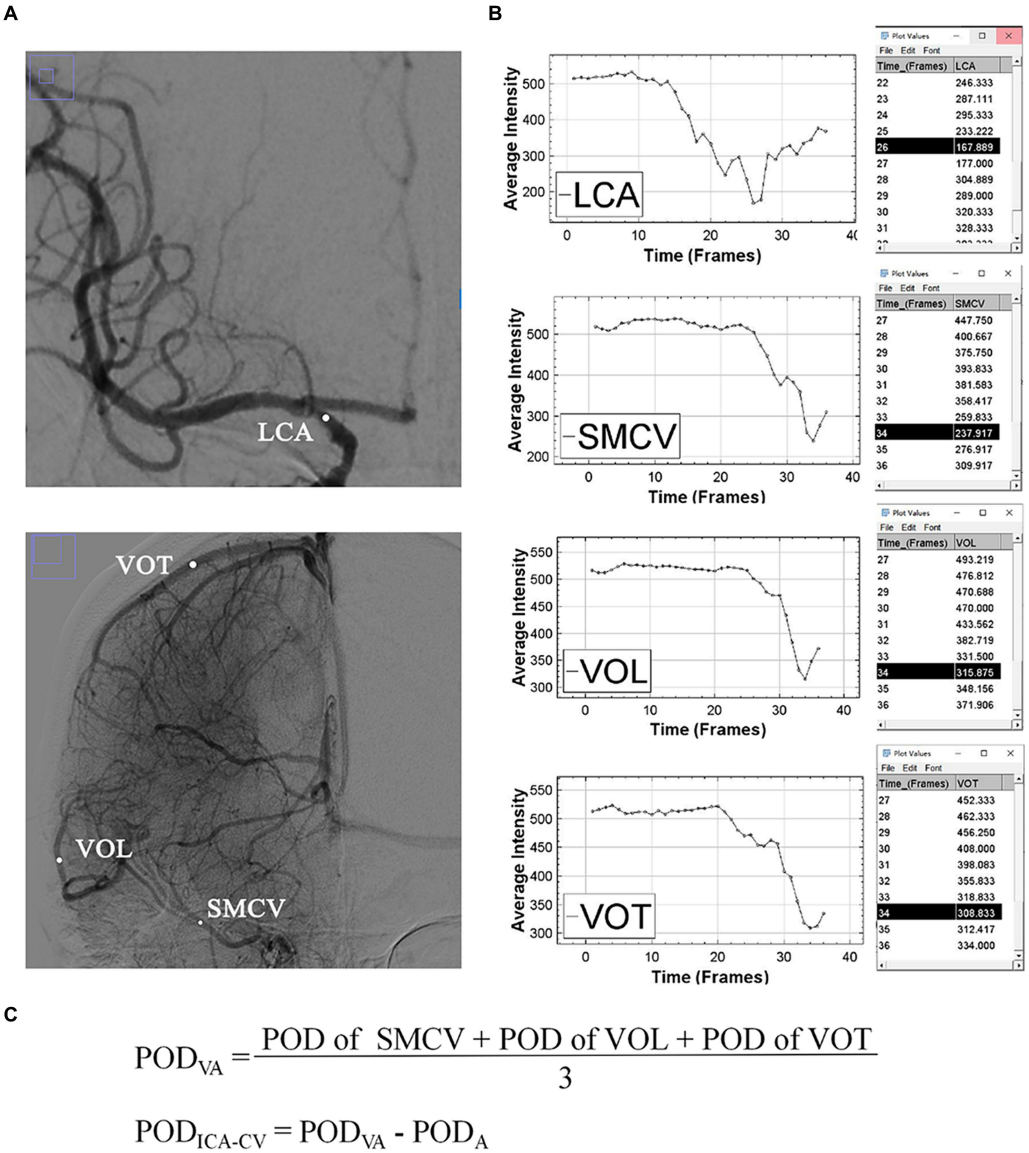
An example of the measurement of POD. **(A)** Shows the regions of interest (white dots) of superficial middle cerebral vein, vein of Labbé and vein of Trolard in the arterial and venous phases. **(B)** Shows the time-density curves and POD values of target vessels. **(C)** Shows the formulas for POD_VA_ and POD_ICA-CV_. ICA, internal carotid artery; SMCV, superficial middle cerebral vein; VOL, vein of Labbé; VOT, vein of Trolard; POD, peak optical density; POD_VA_, cortical venous average POD; POD_A_, POD of terminal internal carotid artery; POD_ICA-CV_, difference between POD_VA_ and POD_A_.

Two-dimensional DSA images including complete venous phase were acquired on an angiographic system (PHILIPS. UNIQ FD20, Holland) at a rate of 6 frames per second in the anterior–posterior and lateral positions separately. On the normal side, 8 mL contrast medium (iodixanol, Yangtze River Pharmaceutical Group, China) was injected with a power injector through a guiding catheter at terminal common carotid artery with 5 mL/s speed. This procedure was uniform in our department. The location of the catheter and injection parameters on the affected side were determined by the interventionalists during operation. The grades of collateral circulation were determined using the American Society of Intervention and Therapeutic Neuroradiology/Society of Interventional Radiology (ASITN/SIR) score based on pre-thrombectomy DSA images. After the final injection of contrast medium, consecutive DSA images were obtained to assess arterial recanalization status using eTICI grade and were processed using ImageJ software (National Institutes of Health, Bethesda, MD, USA). The superficial middle cerebral vein, vein of Trolard, and vein of Labbé were specifically identified as the target veins for analysis because they were dominant in venous drainage from the MCA territory, easily recognizable on radiological images, and have been extensively studied in previous research (7, 9–12). Additionally, the terminal ICA was chosen as the target artery. Circle regions of interest (ROIs) were positioned on these vessels based on following criteria: (1) in anterior–posterior position; (2) with a diameter slightly smaller than the width of the vessels; (3) without other overlapping vessels presenting in corresponding phases; (4) only on main veins; and (5) as close to the midpoint of the target veins as possible. The Peak Optical Density (POD) of each ROI was measured using the Time Series Analyzer V3 plugin. As all DSA images were processed in the original DICOM format and without color inversion, a lower POD value meant higher the density. The venous average POD (POD_VA_) was calculated by adding up the individual POD values of all visible target veins and then dividing this sum by the total number of veins. The difference value of peak optical density between ICA and cortical veins (POD_ICA-CV_) was calculated by subtracting the POD of ICA (POD_A_) from POD_VA_. Figure 2 provided an example of the procedure.

### Outcome measurement

The functional outcomes were evaluated using the mRS scores at 90 days after thrombectomy. In our center, it is standard practice to conduct a prospective follow-up investigation of mRS scores in patients who underwent thrombectomy. The primary outcome was a good functional outcome, defined as an mRS score of 0–2 at 90 days. The secondary outcome was hemorrhagic transformation (HT) observed on follow-up CT or MR images.

### Statistical analysis

The distribution of continuous variables was assessed by the Kolmogorov–Smirnov test. Continuous variables were analyzed using Wilcoxon rank-sum tests, while categorical variables were analyzed using the chi-square test or the Fisher’s exact test. The level of agreement for POD between different observers was measured using the intraclass correlation coefficient (ICC) analysis. A low POD was defined as a POD value lower than the cutoff value determined by receiver operating characteristic curves (ROC) analysis with the mRS score of 0–2 at 90 days as the outcome. The variables associated with good functional outcome, low POD_VA_, low POD_ICA-CV_ and HT in the univariate analyses were adjusted in multivariable logistic regression analyses. Delong tests were performed to compare the predictive abilities of three regression models: (1) model 1 adjusted for age, history of coronary heart disease, ASPECTS, ASITN/SIR score, initial NIHSS score, eTICI grade of 3, HT and infarct volume; (2) model 2 adjusted for the variables in model 1 and ipsilateral POD_VA_; (3) model 3 adjusted for the variables in model 1 and ipsilateral POD_ICA-CV_. Spearman correlation coefficient was used to analyze the associations among ipsilateral POD_ICA-CV_, mRS score at 90 days, eTICI grade, grade of collateral circulation and infarct volume. Mediation analysis using Model 4 in the PROCESS Marco was performed to assess whether the association between the grades of collateral circulation and mRS scores was mediated by POD_ICA-CV_. The number of bootstrap samples was 5,000. Indirect effect was considered significant when 95% CI did not include zero. A *p*-value <0.05 was considered statistically significant. All statistical analyses were performed using SPSS version 25.0 (IBM, Armonk, New York, USA) and MedCalc version 20.218 (MedCalc Software, Ostend, Belgium).

## Results

A total of 488 patients who underwent thrombectomy were reviewed in this study. Finally, a total of 166 patients were included based on inclusion and exclusion criteria. The median (interquartile range [IQR]) age of these patients was 68 (57–73) years and 104 (62.7%) patients were male. The median (IQR) initial NIHSS was 17 (13–21). After thrombectomy, 95 (57.2%) of the patients achieved eTICI grade of 3, 36 (21.7%) of the patients attained an eTICI grade of 2c, while 14.5 and 6.6% achieved eTICI grades of 2b67 and 2b50, respectively. HT and infarct volume were not assessed in 7 patients due to the missing of follow-up images. Overall, 34 (20.5%) of the patients experienced HT and the median (IQR) infarct volume was 14.75 (3.77–61.53) ml. Additionally, 90 (54.2%) of the patients achieved a mRS score of 0–2 at 90 days.

### PODA, PODVA and PODICA-CV

POD data were compared between the two hemispheres in different patients to clarify the differences. Overall, POD was measured in 154 (92.2%) patients for the ipsilateral hemisphere and in 65 (38.9%) patients for the contralateral hemisphere. The reproducibility for POD measurements between observers was found to be excellent. Specifically, the ICC for ipsilateral POD_A_ was 0.928, for ipsilateral POD_VA_ it was 0.901, and for ipsilateral POD_ICA-CV_ it was 0.834. On the contralateral side, the ICC for POD_A_ was 0.938, for POD_VA_ it was 0.892, and for POD_ICA-CV_ it was 0.877.

The contralateral POD_A_ was found to be higher than the ipsilateral POD_A_ (median [IQR], 173.987 [144.813–202.975] vs. 118.668 [84.659–159.686], *p* < 0.001). However, no significant difference was found between the ipsilateral and contralateral POD_VA_ (median [IQR], 278.317 [233.298–311.679] vs. 272.926 [239.371–296.095], *p* = 0.53). Ipsilateral POD_ICA-CV_ was higher than contralateral POD_ICA-CV_ (median [IQR], 150.724 [119.583–186.023] vs. 101.744 [74.941–123.314], *p* < 0.001).

**Figure 3:**
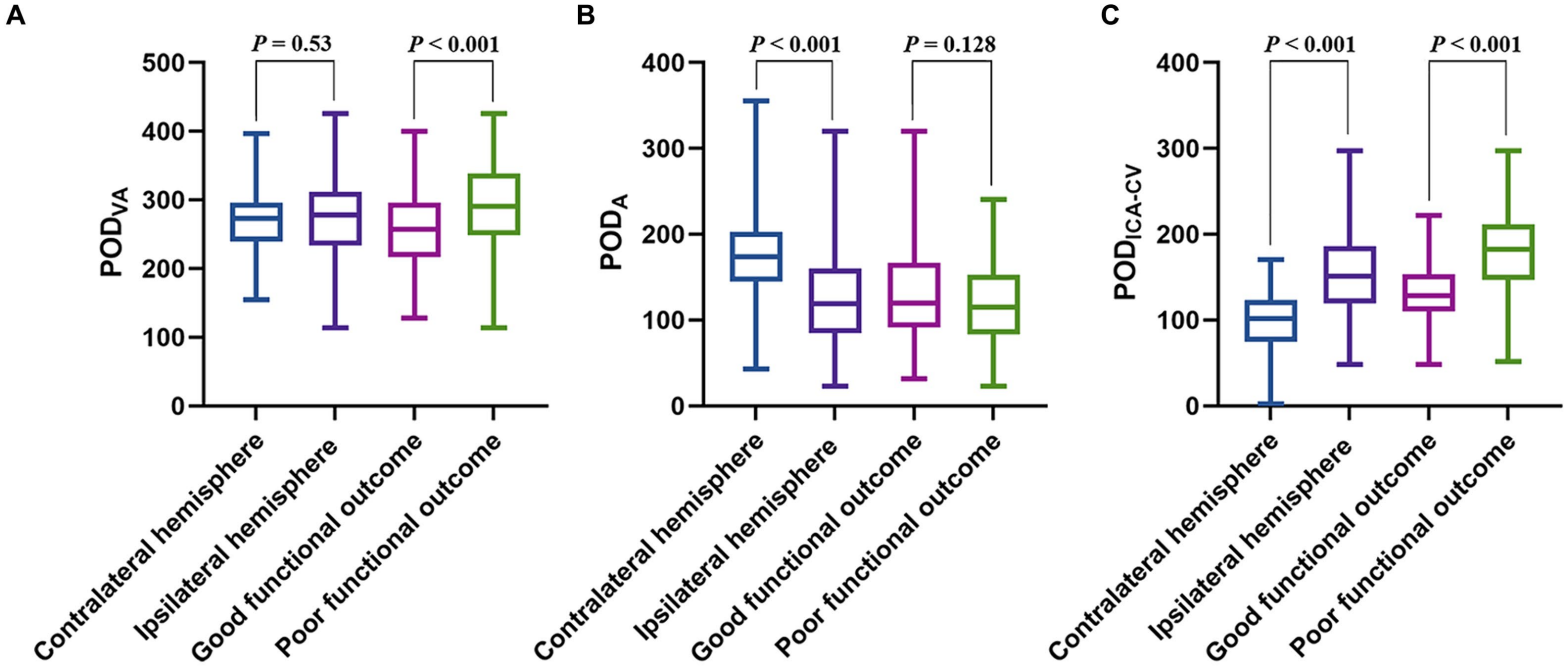
The boxplots show the POD differences among different groups. **(A)** Shows significant difference in ipsilateral POD_VA_ between good and poor functional outcome groups, but no difference between POD_VA_ in different hemispheres. **(B)** Shows significant difference in POD_A_ between bilateral hemispheres, but no difference between ipsilateral POD_A_ in different functional outcome groups. **(C)** Shows significant differences in POD_ICA-CV_ between bilateral hemispheres and ipsilateral POD_ICA-CV_ between good and poor functional outcome groups. POD, peak optical density; POD_VA_, cortical venous average POD; POD_A_, POD of terminal internal carotid artery; POD_ICA-CV_, difference between POD_VA_ and POD_A_.

The baseline characteristics between good and poor functional outcome groups were summarized in Table 1. For the patients with good functional outcomes, both ipsilateral POD_VA_ (median [IQR], 257.198 [216.623–296.631] vs. 290.944 [248.647–338.819], *p* < 0.001) and ipsilateral POD_ICA-CV_ (median [IQR], 128.463 [110.233–153.624] vs. 182.01 [146.621–211.331], *p* < 0.001) were lower than the patients with poor functional outcome. The ipsilateral POD_A_ between good and poor outcome groups was similar (median [IQR], 119.914 [91.619–166.566] vs. 115.239 [83.554–152.824], *p* = 0.128). Compared with the ipsilateral POD_ICA-CV_ in good outcome group, contralateral POD_ICA-CV_ was lower (*p* < 0.001). The comparation of POD are shown in Figure 3.

**Table 1:** Baseline characteristics in patients with ipsilateral POD (comparation between different prognosis).

Variables	Total	mRS of 0–2 at 90 days	mRS > 2 at 90 days	*p* value
	(*n* = 154)	(*n* = 82)	(*n* = 72)	
Demographics				
Male, *n* (%)	95 (61.7)	50 (61)	45 (62.5)	0.846
Age, median (IQR)	68 (58–74)	65 (55–73)	69 (62–75)	0.034
Medical history				
Hypertension, *n* (%)	104 (67.5)	53 (64.6)	51 (70.8)	0.412
Diabetes mellitus, *n* (%)	23 (14.9)	11 (13.4)	12 (16.7)	0.572
Hyperlipidemia, *n* (%)	32 (20.8)	19 (23.2)	13 (18.1)	0.435
Atrial fibrillation, *n* (%)	57 (37)	29 (35.4)	28 (38.9)	0.651
Coronary heart disease, *n* (%)	69 (44.8)	29 (35.4)	40 (55.6)	0.012
Smoking, *n* (%)	46 (29.9)	26 (31.7)	20 (27.8)	0.595
Drinking, *n* (%)	30 (20.8)	15 (18.3)	17 (23.6)	0.417
TOAST classification				0.672
LAA, *n* (%)	83 (53.9)	46 (56.1)	37 (51.4)	
CE, *n* (%)	52 (33.8)	26 (31.7)	26 (36.1)	
Other, *n* (%)	11 (7.1)	7 (8.5)	4 (5.6)	
Unknow, *n* (%)	8 (5.2)	3 (3.7)	5 (6.9)	
Stroke details				
Initial NIHSS, median (IQR)	18 (13–21)	16 (12–19)	20 (15–25)	<0.001
IVT, *n* (%)	26 (16.9)	12 (14.6)	14 (19.4)	0.427
OPT (min), median (IQR)	220 (145–350)	202.5 (143.75–332.5)	240 (151.25–407.5)	0.177
PRT (min), median (IQR)	42 (30–58)	39.5 (26.75–59.5)	43.5 (31–57.75)	0.545
ORT (min), median (IQR)	266 (187–386)	257.5 (184–366.5)	281.5 (195.25–447.5)	0.192
Imaging details				
ASPECTS, median (IQR)	10 (8–10)	10 (8–10)	9 (8–10)	0.004
ASITN/SIR, median (IQR)	2 (1–3)	3 (2–3)	2 (1–3)	<0.001
Infarct volume (ml)*, median (IQR)	15.23 (4.36–64.27)	6.98 (2.89–21.05)	57.56 (16.37–151.49)	<0.001
Hemorrhagic transformation*, *n* (%)	32 (20.8)	12 (14.6)	20 (30.8)	0.019
eTICI				0.059
2b50-	9 (5.8)	3 (3.7)	6 (8.3)	
2b67	21 (13.6)	9 (11)	12 (16.7)	
2c	34 (22.1)	17 (20.7)	17 (23.6)	
3	90 (58.4)	53 (64.6)	37 (51.4)	
Ipsilateral POD_VA_, median (IQR)	278.317 (233.298–311.679)	257.198 (216.623–296.631)	290.944 (248.647–338.819)	<0.001
Ipsilateral POD_A_, median (IQR)	118.668 (84.659–159.686)	119.914 (91.619–166.566)	115.239 (83.554–152.824)	0.128
Ipsilateral POD_ICA-CV_, median (IQR)	150.724 (119.583–186.023)	128.463 (110.233–153.624)	182.01 (146.621–211.331)	<0.001
Low ipsilateral POD_ICA-CV_, n (%)	100 (64.9)	72 (87.8)	28 (38.9)	<0.001

*Follow-up images were missing in 7 cases.

IQR, interquartile range; mRS, modified Rankin Scale; TOAST, the Trial of ORG 10,172 in Acute Stroke Treatment; LAA, large-artery atherosclerosis; CE, cardioembolic; NIHSS, National Institutes of Health Stroke Scale; IVT, intravenous thrombolysis treatment; OPT, onset-to-puncture time; PRT, puncture-to-recanalization time; ORT, onset-to-recanalization time; ASPECTS, Alberta Stroke Program Early CT Score; ASITN/SIR, American Society of Intervention and Therapeutic Neuroradiology/Society of Interventional Radiology; eTICI, expanded Thrombolysis in Cerebral Infarction; POD_VA_, cortical venous average peak optical density; POD_A_, peak optical density of terminal internal carotid artery; POD_ICA-CV_, difference between POD_VA_ and POD_A_.

### Association between POD and functional outcomes

Table 2 summarizes the results of univariate and multivariable analyses. After adjusting for age, history of coronary heart disease, ASPECTS, ASITN/SIR score, initial NIHSS score, eTICI grade of 3, HT and infarct volume related to good functional outcome with *p* < 0.1, multivariable logistic regression analyses showed that both ipsilateral POD_VA_ (odds ratio [OR] 0.991, 95% confidence interval [CI] 0.984–0.999, *p* = 0.019) and ipsilateral POD_ICA-CV_ (OR 0.975, 95% CI 0.963–0.986, *p* < 0.001) were significantly associated with good functional outcome. Moreover, ipsilateral POD_A_ was not an independent predictor of good functional outcome (OR 1.006, 95% CI 0.998–1.015, *p* = 0.137). Ipsilateral POD_ICA-CV_ was positive correlated with mRS score at 90 days (*r* = 0.456, *p* < 0.001).

**Table 2:** Univariable and multivariable logistic regression analyses for good functional outcome (mRS of 0–2 at 90 days).

Independent variables	Good functional outcome			
	Unadjusted OR (95% CI)	*p* value	Adjusted OR (95% CI)	*p* value
Ipsilateral POD_VA_	0.99 (0.984–0.996)	<0.001	0.991 (0.984–0.999)	0.019
Ipsilateral POD_A_	1.005 (0.999–1.012)	0.087	1.006 (0.998–1.015)	0.137
Ipsilateral POD_ICA-CV_	0.974 (0.965–0.984)	<0.001	0.975 (0.963–0.986)	<0.001

The multivariable regression model was adjusted for age, history of coronary heart disease, ASPECTS, ASITN/SIR score, initial NIHSS score, eTICI grade of 3, hemorrhagic transformation and infarct volume. OR, odds ratio; CI, confidence interval; mRS, modified Rankin Scale; POD_VA_, cortical venous average peak optical density; POD_A_, peak optical density of terminal internal carotid artery; POD_ICA-CV_, difference between POD_VA_ and POD_A_; ASPECTS, Alberta Stroke Program Early CT Score; ASITN/SIR, American Society of Intervention and Therapeutic Neuroradiology/Society of Interventional Radiology; NIHSS, National Institutes of Health Stroke Scale; eTICI, expanded Thrombolysis in Cerebral Infarction.

### Predictive ability for good functional outcome

The predictive ability for good functional outcomes was assessed using area under the curve (AUC). The AUC for ipsilateral POD_VA_ was 0.667 (95% CI 0.581–0.753, *p <* 0.001) with the cutoff value of 259.915, sensitivity of 73.6% and specificity of 53.7%. While the AUC for ipsilateral POD_ICA-CV_ was 0.787 (95% CI 0.713–0.861, *p* < 0.001) with the cutoff value of 163.088, sensitivity of 61.1% and specificity of 87.8%.

**Figure 4:**
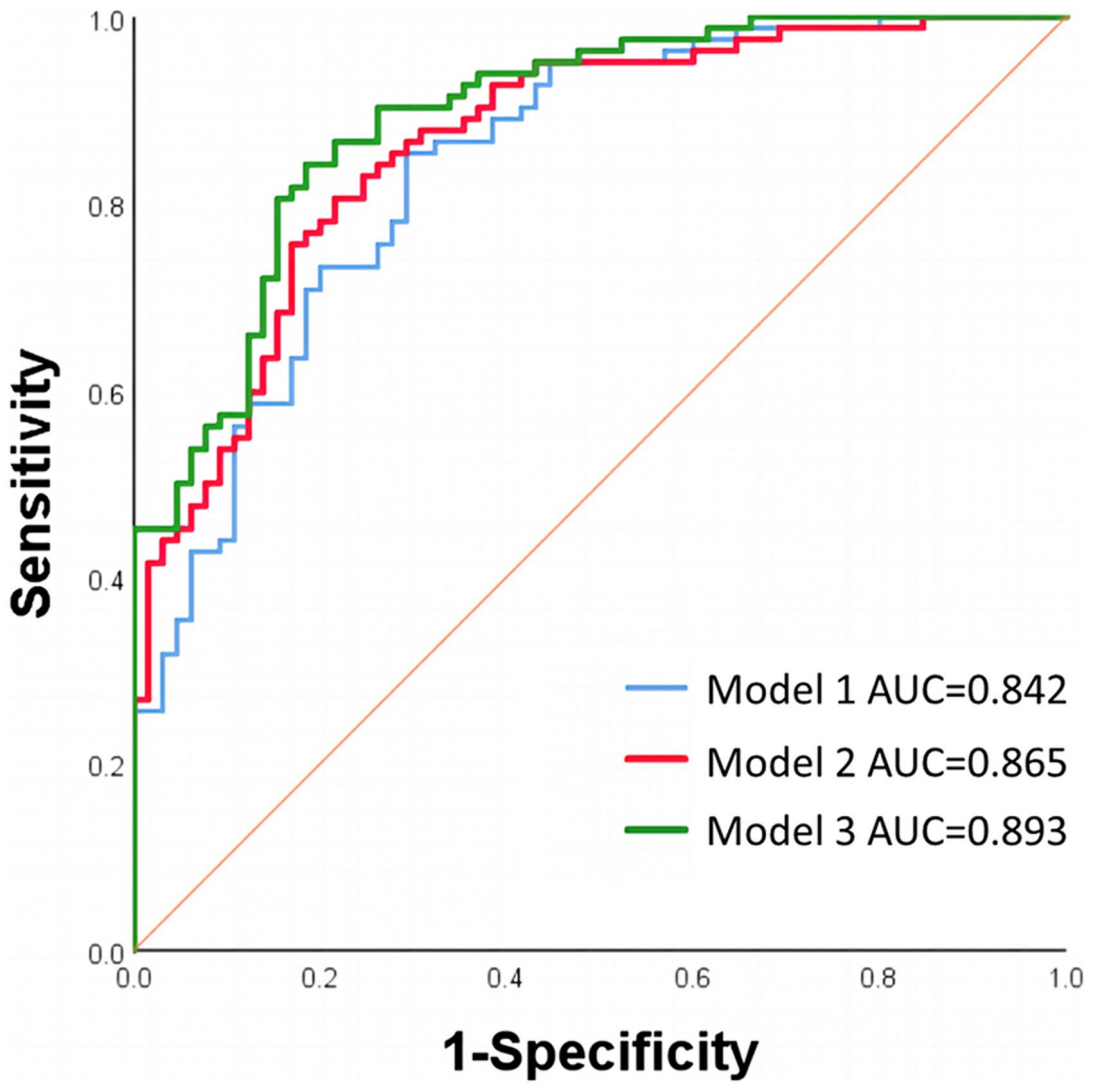
ROCs of different regression models. Model 1 was adjusted for age, history of coronary heart disease, ASPECTS, ASITN/SIR score, initial NIHSS score, eTICI grade of 3, hemorrhagic transformation and infarct volume. Model 2 was adjusted for the variables in model 1 and ipsilateral POD_VA_. Model 3 was adjusted for the variables in model 1 and ipsilateral POD_ICA-CV_. ROCs, receiver operating characteristic curves; AUC, area under the curve; ASPECTS, Alberta Stroke Program Early CT Score; ASITN/SIR, American Society of Intervention and Therapeutic Neuroradiology/Society of Interventional Radiology; NIHSS, National Institutes of Health Stroke Scale; eTICI, expanded Thrombolysis in Cerebral Infarction; POD_VA_, cortical venous average peak optical density; POD_ICA-CV_, difference between POD_VA_ and peak optical density of terminal internal carotid artery.

When including ipsilateral POD_ICA-CV_ in the baseline model, there was a significant increase in AUC (0.893 vs. 0.842, *p* = 0.027). Compared with baseline model, the enhancement of predictive ability of model 2 adjusted for ipsilateral POD_VA_ and the variables in baseline model was not significant (0.865 vs. 0.842, *p* = 0.107). The ROCs comparation were shown in Figure 4.

### Association with collateral circulation and eTICI grade

In 154 patients with ipsilateral POD, 63 (40.9%) of them had low POD_VA_ and 100 (64.9%) of them had low POD_ICA-CV_. Both ASITN/SIR score (OR 1.183, 95% CI 0.907–1.543, *p* = 0.216) and eTICI grade (OR 0.831, 95% CI 0.588–1.177, *p* = 0.298) had no relationship with low ipsilateral POD_VA_ in univariate analysis. After adjusted for age, initial NIHSS score and eTICI grade, it was found that only the ASITN/SIR score was significantly associated with low ipsilateral POD_ICA-CV_ (OR 1.391, 95% CI 1.023–1.891, *p =* 0.035) in multivariable logistic regression analysis. This founding was shown in Table 3. Moreover, there was a negative correlation between ipsilateral POD_ICA-CV_ and ASITN/SIR score (*r* = −0.193, *p* = 0.016). No linear relationship was found between eTICI grade and POD_ICA-CV_ (*r* = 0.133, *p* = 0.099).

**Table 3:** Multivariable logistic regression analysis for low ipsilateral POD_ICA-CV_.

Independent variables	Ipsilateral POD_ICA-CV_ < 163.088	
	Adjusted OR (95% CI)	*p* value
Age	0.983 (0.951–1.017)	0.321
ASITN/SIR	1.391 (1.023–1.891)	0.035
Initial NIHSS	0.95 (0.901–1.002)	0.061
eTICI	1.198 (0.823–1.745)	0.346

OR, odds ratio; CI, confidence interval; POD_ICA-CV_, difference value of peak optical density between internal carotid artery and cortical veins; ASITN/SIR, American Society of Intervention and Therapeutic Neuroradiology/Society of Interventional Radiology; NIHSS, National Institutes of Health Stroke Scale; eTICI, expanded Thrombolysis in Cerebral Infarction.

### Mediation effect of PODICA-CV

**Figure 5:**
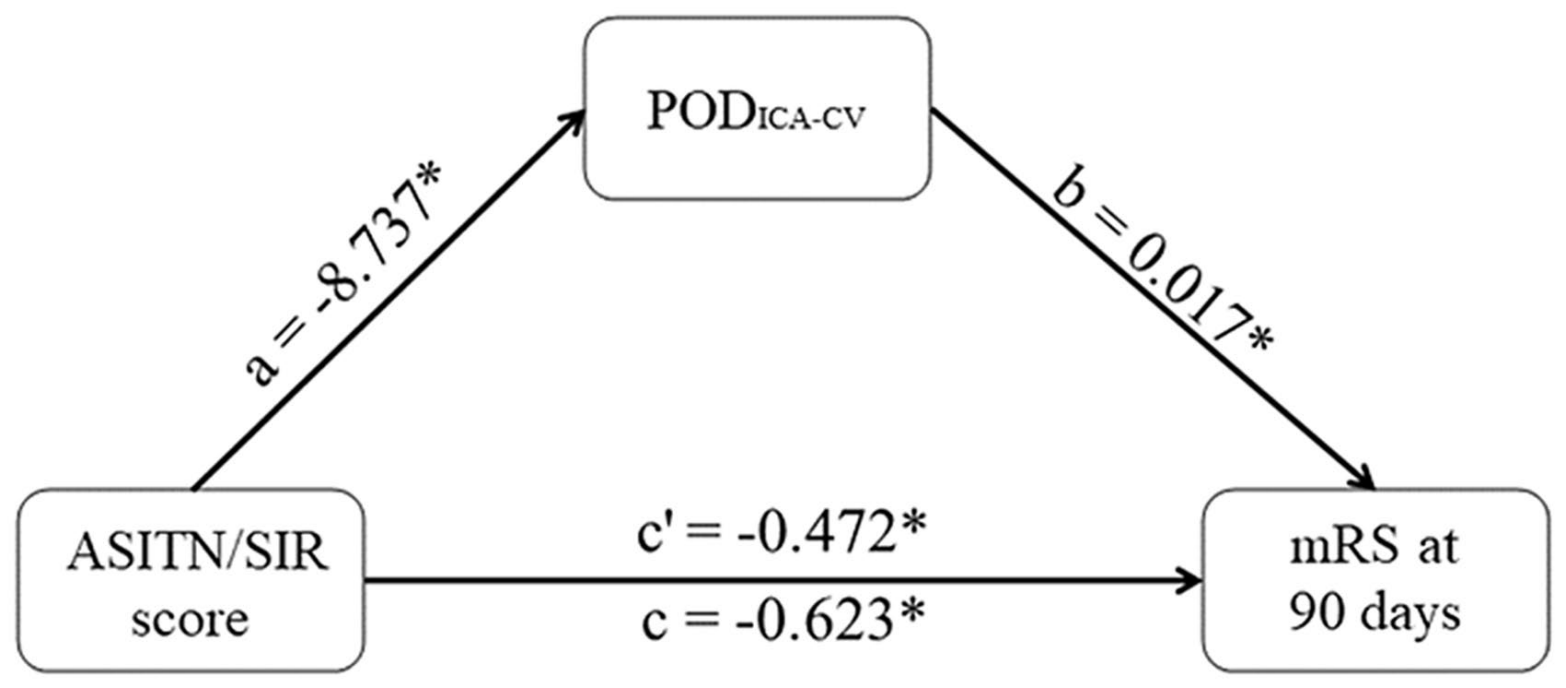
Mediation effect of POD_ICA-CV_. POD_ICA-CV_ partly mediates the effects of ASITN/SIR scores on mRS at 90 days. The coefficient c is the total effect and c’ is the direct effect. All coefficients are unstandardized. *p < 0.05. POD_ICA-CV_, difference between POD_VA_ and peak optical density of terminal internal carotid artery; ASITN/SIR, American Society of Intervention and Therapeutic Neuroradiology/Society of Interventional Radiology; mRS, modified Rankin Scale.

The effects of ASITN/SIR scores on POD_ICA-CV_ (*β* = −0.209, *p* = 0.093) and POD_ICA-CV_ on mRS scores (*β* = 0.401, *p* < 0.001) were statistically significant in the mediation analysis. Partial mediation effect of POD_ICA-CV_ on the relationship between the collateral circulation and mRS scores was confirmed, accounting for 24.2% of the total effect (indirect effect −0.151, 95% CI −0.282–−0.034; total effect −0.623, 95% CI −0.893–−0.352). These results are shown in Figure 5.

### Relationship with HT and infarct volume

Ipsilateral POD_VA_ was not associated with HT in univariate analysis (OR 1.001, 95% CI 0.995–1.008, *p =* 0.715). Table 4 shows a significant association between ipsilateral POD_ICA-CV_ and HT (OR 1.008, 95% CI 1.0004–1.016, *p =* 0.04) after adjusting for diabetes mellitus history. A positive correlation was found between ipsilateral POD_ICA-CV_ and infarct volume (*r* = 0.361, *p* < 0.001).

**Table 4:** Multivariable logistic regression analysis for hemorrhagic transformation.

Independent variables	Hemorrhagic transformation	
	Adjusted OR (95% CI)	*p* value
Diabetes mellitus	0.169 (0.022–1.322)	0.09
Ipsilateral POD_ICA-CV_	1.008 (1.0004–1.016)	0.04

OR, odds ratio; CI, confidence interval; POD_ICA-CV_, difference value of peak optical density between internal carotid artery and cortical veins.

## Discussion

In our study, we conducted a novel approach to quantify the VO on DSA images and reflect the status of microcirculation and venous circulation after successful arterial recanalization which may involve in the “non-reflow” phenomenon (6). We found that the POD_A_ taken as the background value showed no difference in patients with different prognosis, but the higher ipsilateral POD_VA_ and higher ipsilateral POD_ICA-CV_ were more likely to occur in poor functional outcome group. Both ipsilateral POD_VA_ and ipsilateral POD_ICA-CV_ were independent predictors of functional outcomes. Furthermore, contralateral POD_ICA-CV_ was significantly less than ipsilateral POD_ICA-CV_. Our findings suggest that POD is an effective DSA parameter for assessing cerebral circulatory function and further demonstrate that the importance of VO in the postoperative evaluation.

VO is under the influence of upstream circulatory interaction. In animal stroke models, the reduction of VO has been observed following the occlusion of MCA, and animals with poor collaterals are more likely to have complete absence of VO which is associated with severe functional deficits and larger final infarction (13). The VO changes can also be observed on CTA images in AIS-LVO patients. This CTA VO changes is related with the ratio of brain tissue with Tmax >10 s/Tmax >6 s which is an important indicator for cerebral microcirculatory perfusion (7). With the increasing of the related researches, the pre-thrombectomy VO has been considered as a strong predictor of successful arterial recanalization, clinical outcomes and brain edema (14–16). As a result, evaluating pre-thrombectomy VO can provide a more precise information for identifying patients who would benefit from thrombectomy, thereby preventing unnecessary surgical procedures and the development of complications. This is particularly important in the cases that perfusion imaging is not available.

After thrombectomy, the abnormal VO usually represents poor cerebral reperfusion. For instance, luxury perfusion, a condition characterized by early venous filling, has been observed to frequently occur after thrombectomy and can lead to HT (17). However, quantifying premature venous filling is difficult and its effect on functional outcomes is still a matter of debate. Only one study has successfully used a quantitative method to evaluate postoperative VO by measuring the TTP of rolandic vein and has shown that a shorter TTP difference between distal MCA and rolandic vein is a marker of sufficient and effective microcirculatory reperfusion (8). This finding highlights the importance of evaluating both arterial and venous features in assessing cerebral circulation, rather than focusing solely on arteries or veins. In our study, we further confirmed this finding by comparing the AUCs of different regression models using the Delong test. We found that including ipsilateral POD_ICA-CV_ in the baseline model significantly improved its predictive ability compared to including ipsilateral POD_VA_. Furthermore, different from the previous study that investigated only a single cortical vein (8), we evaluated a wider range from the ICA to three dominant cortical veins, resulting in the ipsilateral POD_ICA-CV_ becoming a more comprehensive indicator of cerebral reperfusion and providing an additional potential for predicting HT. This insight is supported by a recent study that demonstrates persistent dysfunction between the ICA and distal small vessels, even with a mTICI score of 2b-3 after thrombectomy (18).

Collateral circulation is considered as a crucial factor in determining outcomes of patients with acute ischemic stroke (19). Even in the late time window, robust collaterals can prevent stroke patients from the progression of ischemic core and severe functional deficits (20). The AIS-LVO patients with robust collaterals always have a superior response to thrombectomy and better outcomes (21). In our study, we found that collateral circulation was the only independent predictor of low ipsilateral POD_ICA-CV_, and patients with lower ipsilateral POD_ICA-CV_ were more likely to have robust collaterals, small infarct volume, and better functional outcomes. The relationship between grades of collateral circulation and mRS scores was partially mediated by POD_ICA-CV_ (mediation proportion 24.2%). As a functional indicator of cerebral circulation, ipsilateral POD_ICA-CV_ may be helpful to explain the patients with good collateral circulation but poor prognosis after stroke.

Unlike previous studies (8), our study yielded different findings as it did not observe any impact of expanded recanalization grade on prognosis. Although a higher proportion of eTICI grade of 3 was found in favorable prognosis patients, it did not predict outcomes in multivariate analysis. Similarly, the effect of various expanded recanalization grades on ipsilateral POD_ICA-CV_ was also not detected. Moreover, we identified that both ipsilateral POD_ICA-CV_ and infarct volume, which was a crucial influential factor of outcomes (22), served as independent predictors of outcome. Our results provide additional insight into the notion that the “non-reflow” phenomenon cannot be solely attributed to incomplete recanalization (6).

Quantitative characteristics of cerebral circulation on DSA images have been successfully utilized to predict the stroke risk in the patients (18), hyperperfusion after carotid artery stenting or angioplasty (23), post-thrombectomy HT (24) and clinical outcomes (8, 25, 26). Nevertheless, there is no standardized method to quantify these characteristics. In the balloon test occlusion study, the DSA frame numbers of the starting of venous phases were recorded to analyze the difference between bilateral cerebral circulation (27). The similar frame numbers counting method was adopted in two previous studied to measure cerebral circulation time (18, 28). Additionally, there are several studies using a variety of software to quantify cerebral circulatory function (8, 24, 25, 29, 30). Most of the methods have limitation of generalizability because of the non-open source software or high technical barrier. To better evaluate the cerebral circulatory function quantitatively, our study performed open-source ImageJ software to process DSA images. ImageJ is a free and open-source image processing software primarily used for scientific research and biomedical fields. It offers a range of powerful image processing and analysis tools available to measure and segment a stack of dynamic images. As a parameter of quantified DSA, the vascular peak density is investigated rarely (31). However, Liu et al. (25) pointed out that it has an advantage over TTP in predicting outcomes after thrombectomy. For this reason, we used POD to assess VO and the function of cerebral circulation.

Our study has several limitations. First, it was a retrospective single-center design with a relatively small sample size, which may introduce selection bias. Second, due to the small number of patients with complete contralateral venous phase on DSA images, we could not use the method referred in the previous study to measure cerebral circulation time and compare it with our parameters. Third, our method for evaluating VO cannot be applied in patients whose target veins are not opacified after thrombectomy. The absence of venous opacification is usually caused by the failed arterial recanalization or severe obstruction in downstream vessels. Our results should be confirmed in the prospective intervention trials in the future.

## Conclusion

The ipsilateral POD_ICA-CV_ easily acquired from DSA images reflected more comprehensive cerebral circulatory function. It was associated with function outcomes after thrombectomy and proposed as an indicator to assess effectiveness of thrombectomy. The further reduction of ipsilateral POD_ICA-CV_ after successful arterial recanalization may lead to a better prognosis in AIS-LVO patients.

## Data availability statement

The original contributions presented in the study are included in the article/supplementary material, further inquiries can be directed to the corresponding authors.

## Ethics statement

The studies involving humans were approved by the Institutional Review Board of Xiangyang No.1 People’s Hospital, Hubei University of Medicine. The studies were conducted in accordance with the local legislation and institutional requirements. The ethics committee/institutional review board waived the requirement of written informed consent for participation from the participants or the participants’ legal guardians/next of kin due to the retrospective nature of the study.

## Author contributions

GL: Conceptualization, Formal analysis, Methodology, Software, Validation, Writing – original draft. JC: Data curation, Methodology, Software, Writing – original draft. PZ: Investigation, Methodology, Validation, Writing – original draft. DS: Formal analysis, Investigation, Writing – original draft. ZK: Formal analysis, Validation, Writing – original draft. RF: Investigation, Writing – original draft. BM: Funding acquisition, Methodology, Project administration, Software, Validation, Writing – review & editing. JZ: Conceptualization, Project administration, Supervision, Visualization, Writing – review & editing.
